# Elucidating the mechanisms of cooperative calcium-calmodulin interactions: a structural systems biology approach

**DOI:** 10.1186/1752-0509-2-48

**Published:** 2008-06-02

**Authors:** Najl V Valeyev, Declan G Bates, Pat Heslop-Harrison, Ian Postlethwaite, Nikolay V Kotov

**Affiliations:** 1 Systems Biology Lab, Department of Engineering, University of Leicester, University Road, Leicester, LE1 7RH, UK; 2 Systems Biology Lab, Department of Biology, University of Leicester, University Road, Leicester, LE1 7RH, UK; 3 Biophysics & Bionics Lab, Department of Physics, Kazan State University, Kazan 420008, Russia

## Abstract

**Background:**

Calmodulin is an important multifunctional molecule that regulates the activities of a large number of proteins in the cell. Calcium binding induces conformational transitions in calmodulin that make it specifically active to particular target proteins. The precise mechanisms underlying calcium binding to calmodulin are still, however, quite poorly understood.

**Results:**

In this study, we adopt a structural systems biology approach and develop a mathematical model to investigate various types of cooperative calcium-calmodulin interactions. We compare the predictions of our analysis with physiological dose-response curves taken from the literature, in order to provide a quantitative comparison of the effects of different mechanisms of cooperativity on calcium-calmodulin interactions. The results of our analysis reduce the gap between current understanding of intracellular calmodulin function at the structural level and physiological calcium-dependent calmodulin target activation experiments.

**Conclusion:**

Our model predicts that the specificity and selectivity of CaM target regulation is likely to be due to the following factors: variations in the target-specific Ca^2+ ^dissociation and cooperatively effected dissociation constants, and variations in the number of Ca^2+ ^ions required to bind CaM for target activation.

## Background

Calmodulin (CaM) is a multisite and multifunctional protein that contains four EF-hand Ca^2+ ^binding sites [1], and is involved in a wide variety of cellular functions [2]. For example, it regulates the concentration of intracellular cAMP concentration in a very complex manner by regulating activities of cAMP producing adenylate cyclases (AC) and cAMP hydrolysing enzyme phosphodiesterase (PDE). CaM also regulates a large number of kinases and phosphatases as well as other enzymes with opposing cellular effects. Despite a large number of experimental studies [3-15], the detailed mechanisms underlying CaM-dependent intracellular regulation of such a large variety of target proteins are still not fully understood.

**Figure 1:**
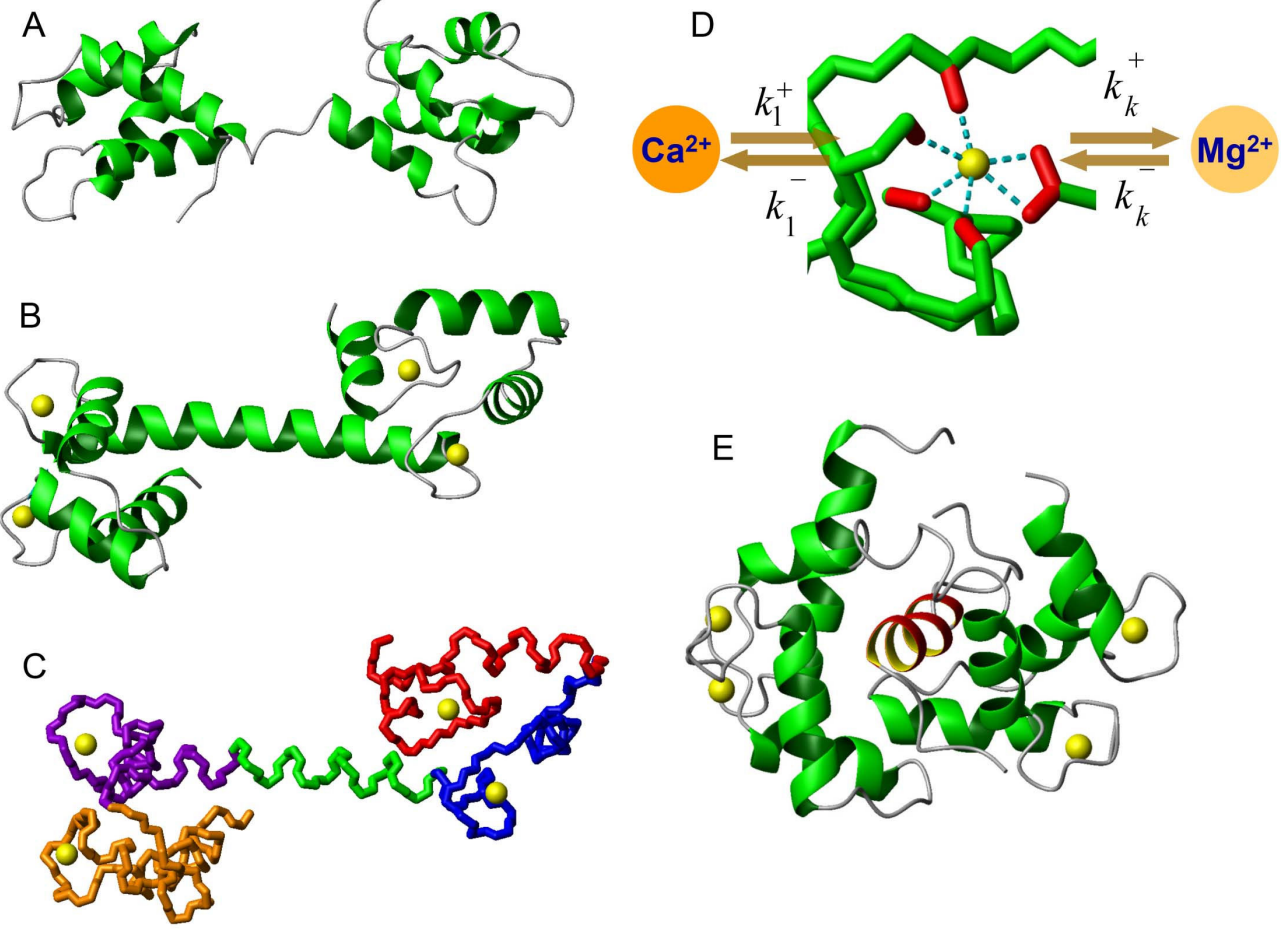
**The analysis of the CaM structures in apo and Ca^2+ ^bound conformations as well as in complex with the target PKII molecule**. The apo (1DMO [18]) (A) and Ca^2+ ^bound (1CLL [19]) (B) structures of the CaM protein illustrate Ca^2+ ^induced conformational transitions. (C) The backbone representation of the calcium bound form (1CLL [19]) highlights the pairwise proximity of the (red and blue, violet and brown) Ca^2+ ^binding EF hand domains and at the same time raises doubts about overall cooperativity between binding sites. (D) Structure of the Ca^2+ ^loop on the N-terminal of the CaM protein (1CLL [19]). The dotted line shows the interaction between oxygen atoms on the sidechains of Asp20, Asp22, Asp24, Thr26, Glu31 residues and Ca^2+ ^or Mg^2+ ^ions. (E) The ribbon diagram of the Ca^2+^-CaM in complex with the target peptide of the protein kinase kinase (1CKK [22]).

Previous investigations of CaM structures in complexes with target protein peptides have led to the view that the specificity in CaM-dependent target activation arises from the diversity of interaction interfaces between CaM and its target proteins [16,17]. There are two globular domains in CaM, each containing a pair of helix-loop-helix Ca^2+^-binding motifs called EF-hands (Figure 1A, Figure 1B). Ca^2+ ^binding alters CaM conformation (compare the structure of Ca^2+^-free CaM [18] in Figure 1A with that of fully Ca^2+ ^bound CaM [19] in Figure 1B) and changes its affinity to target proteins. The four helix-loop-helix EF-hand Ca^2+^-binding sites (shown in different colours in Figure 1C) are also capable of binding intracellular calcium's physiologically inactive competitor Mg^2+ ^(Figure 1D) [20,21]. The best known canonical binding mode of CaM interaction with a target is the "wrap-around" in which both *N*- and *C*-terminal domains of CaM bind to the same target protein region. This interaction is exemplified by a CaM structure in complex with the protein kinase kinase peptide [22] shown in Figure 1E. A variation of the wrap-around CaM binding interaction has been shown to occur with the brain specific PKC substrate CAP-23/NAP-22 [23]. Examples of the so-called "extended" mode of interaction include anthrax exotoxin, Edema Factor [6] and Ca^2+ ^Pump [5]. The extended mode of interaction is proposed to occur in target binding to apo-CaM via the IQ motif [24] (also reviewed in [25]). CaM-induced target dimerization has been reported as having another distinctly different binding mode. The dimerization of Ca^2+^-dependent K^+ ^channels is achieved by adopting the extended conformation of the EF-hand domains of CaM protein [26,27]. Two other reported examples of CaM target proteins dimerizing upon interaction are the glutamate decarboxylase (GAD) [28] and transcription factor SEF2-1/E2-2 [29,30].

Recent experimental studies have attempted to elucidate the mechanisms underlying Ca^2+^/CaM-dependent target regulation by measuring the kinetics and steady-state levels of CaM-target binding [3,11,31-33] as well as by analysing the mechanisms of Ca^2+^-CaM interactions [9,12,34-37]. Ca^2+ ^ion binding to EF-hand sites was shown to lead to CaM conformational alterations [1,38-41]. In the modified conformational state, CaM is likely to alter its affinity to different targets by increasing and decreasing its affinity to certain proteins. Ca^2+ ^ion binding to CaM is also argued to positively modulate the affinity of other Ca^2+ ^binding sites of the molecule. There is still, however, an ongoing debate about the existence, the mechanisms and the degree of cooperativity in Ca^2+^-CaM interactions. In some studies, Ca^2+ ^binding to CaM has been reported to be independent [15,37]. On the other hand, other studies have reported cooperative interactions between the neighbouring EF-hand binding sites [12,35] or cooperativity linking all sites of the CaM molecule [9].

A number of previous studies have attempted to use mathematical modelling to obtain a quantitative understanding of the mechanisms involved in Ca^2+^-CaM interactions. Different mathematical models, including the well known Hill [42], Adair [43] and Monod-Wyman-Changeux (MWC) [44] models, have been used in the literature to describe the cooperativity of ligand binding to a multisite protein. The Hill equation is frequently used to qualitatively measure the degree of cooperativity in multisite binding. It describes the simultaneous binding of *n *ligand molecules to a protein where the parameter *n *can be interpreted as the number of bound molecules. The Adair model represents ligand-protein interactions in terms of successive binding steps. The MWC model is based on two conformations that are in equilibrium and have different affinities for a ligand. To date, the Hill and Adair models have been most frequently used to investigate Ca^2+ ^binding to CaM [4,9-12,14,33,45]. While these studies have provided much useful information, the use of the classical models mentioned above also introduces some limitations in the analysis – see [46] for a full discussion of this issue. In particular, the detailed analysis of ligand-protein interactions which are unique to CaM requires the development of a model that captures multiple functionally important intermediate conformations of the protein. A steady-state solution to the cooperativity problem for Ca2+ binding has been analysed in [47]. In this paper, we develop a new model, based on the assumption that the specificity in CaM target regulation arises from the Ca^2+^-CaM complex specific target interactions with variable numbers of bound Ca^2+ ^ions. In this approach, Ca^2+ ^binding to each EF-hand sites causes conformational transitions in the CaM molecule leading to a model that has multiple conformational states in complex with variable numbers of Ca^2+ ^ions. In the proposed model, CaM may regulate its targets with one, two or three Ca^2+ ^ions as well as in the apo- or fully bound states. In particular, we address the Ca^2+^-CaM interaction in significant detail, although we do not incorporate detailed Ca^2+^-CaM species interactions with target proteins. This approach is in agreement with recent experimental evidence that the concentration-dependent profiles for several Ca^2+^-CaM-dependent protein targets exhibit quite a diverse range of behaviour. PMCA and PDE protein concentrations in the active state, for example, reveal "Hill-shape"-like curves, whereas the ACII isoform is inhibited by increasing Ca^2+ ^concentration. The ACVI isoform exhibits inhibition with an interesting plateau feature on the Ca^2+^-dependent profile. Yet ACI isoforms have bell-shaped concentration-dependent profiles [48]. It has also been shown that CaMPKII [49] as well as the K^+ ^channel from Paramecium [50] are activated by CaM with two bound Ca^2+ ^ions.

The structural systems biology approach, [51], employed in this paper provides new insights into the Ca^2+^-CaM-target binding dose-response curves which have been derived experimentally, and allows us to advance testable hypotheses about the nature of cooperative mechanisms unique to calcium-CaM interactions. The resulting analysis further bridges the gap between our understanding of CaM structural properties and intracellular Ca^2+^-CaM-dependent target regulation.

## Results

### Multisite binding of cooperatively linked binding centres

**Figure 2:**
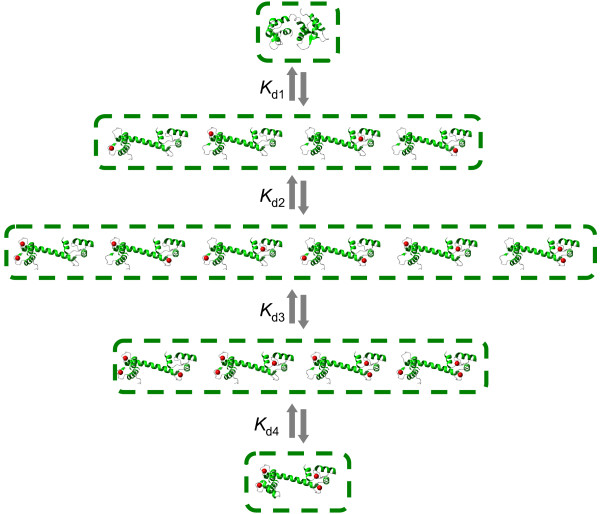
**The kinetic scheme for the non-cooperative model**. **Model 1: **This simplified model is based on the assumption that all Ca^2+ ^molecules are independent of each other and of interactions with a target protein. The relative simplicity of the model allows a straightforward description of CaM-dependent target protein regulation and lays the groundwork for a more complete understanding of cooperative- and target interaction-dependent effects.

**Figure 3:**
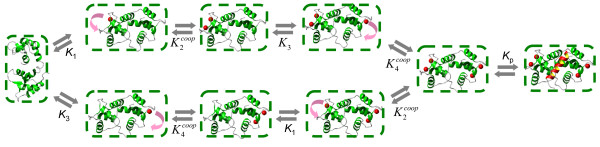
**The kinetic scheme for the intermediate cooperative model**. **Model 2**: This intermediate model extends **Model 1 **to include the effects of Ca^2+^-binding cooperativity. In this model, the first Ca^2+ ^occupied site is assumed to influence and increase the affinity of the second site. The second site, in turn, increases the affinity of the third Ca^2+ ^binding site. The process continues until a CaM molecule is fully bound. This level of modelling allows a qualitative comparison of the concentration profiles for apo-, intermediate- and fully bound Ca^2+^-CaM species with and without cooperative binding as shown on Figure 5.

**Figure 4:**
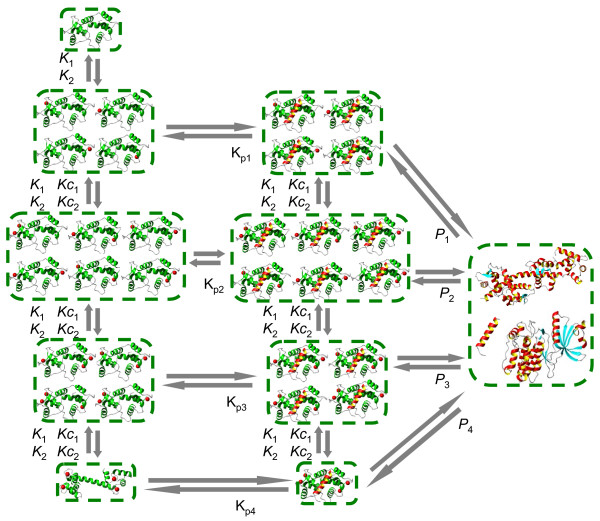
**The kinetic scheme for the full cooperative model**. **Model 3**: This model provides the most realistic description of the Ca^2+^-CaM-target protein complex assembly. The model assumes that both the *N*- and *C*-terminal contain two cooperatively bound EF-hand Ca^2+^-binding pairs. These sites cooperatively influence each other only and do not have any effect on Ca^2+ ^binding on the other terminal. This cooperative interaction is symmetrical, in the sense that any unoccupied site in the terminal increases the affinity of its neighbour in the same way that it would be influenced by its neighbour if its neighbour bound a Ca^2+ ^ion first. Both the "original" and cooperatively influenced dissociation constants are dependent on whether or not CaM is bound to a target protein.

**Figure 5:**
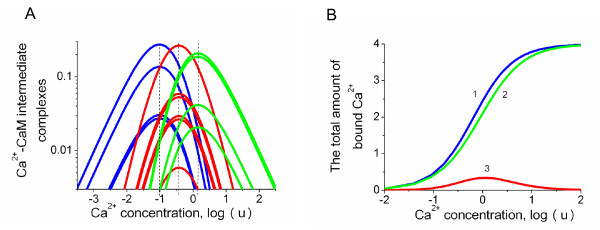
**The effects of cooperative Ca^2+ ^binding to intermediate conformations of CaM**. (A) The log-log graph reveals that cooperativity slightly shifts the positions of the maximum values of the intermediate conformations. The differences in the amount of bound ligand allow quantification of the degree of cooperativity. (B) The total amount of ligand bound to CaM in the presence (1) and absence (2) of cooperative binding. The line (3) shows the difference in the level of bound ligand between the two types of binding mechanisms.

Perhaps one of the most intriguing questions about the intracellular function of CaM is the mechanism and consequences of EF-hand Ca^2+ ^binding site influences leading to cooperative effects. The structure of the CaM molecule (Figure 1A,B,C) has a "dumbbell" shape with two globular domains, each containing two EF-hand Ca^2+ ^binding sites located in the vicinity of each other. These neighboring sites are very likely to structurally influence each other upon Ca^2+ ^binding to one of them. On the other hand, there does not seem to be a direct mechanism to "transfer" the structural alterations between the *N*- and *C*-terminal binding sites upon ion binding. It is, therefore, reasonable to assume that the EF-hand Ca^2+ ^binding sites on CaM are subject to pairwise influence. In order to explore how cooperative mechanisms and CaM-target protein interactions influence Ca^2+ ^binding to CaM, we developed three models with increasing levels of complexity (**Models 1, 2 **and **3 **in Materials and Methods) that are illustrated schematically in Figures 2, 3 and 4, respectively. **Model 1 **disregards the cooperativity between the Ca^2+ ^binding sites (Figure 3). **Model 2 **is an intermediate model which extends the previous representation by introducing the cooperative dependence of one site on another in each domain. A comparison of the concentration profiles generated by **Model 1 **and **Model 2 **clearly reveals the impact of cooperativity on Ca^2+ ^binding to CaM. However, **Model 2 **is still incomplete as it does not capture the reverse or symmetrical cooperative dependence, when one Ca^2+ ^binding site cooperatively influences another, and another site can in the same way influence the first site, within both terminals. To address this issue, a third model, **Model 3, **is developed to incorporate described cooperative influence of EF-hand Ca^2+ ^binding sites (Figure 5).

In order to investigate the dependence of one Ca^2+ ^binding site on another in both the *N*- and *C*-terminal domains, we assumed the alteration of a dissociation constant when a neighboring site is occupied as illustrated schematically in Figure 3 (compare the **Model 1 **and **Model 2 **descriptions in the Materials and Methods section). This approximation allows the derivation of a model that has an analytical solution in the form of conditional probabilities (Equation 12). It provides a quantitative comparison of the concentration of Ca^2+ ^bound to CaM in the presence and absence of Ca^2+ ^binding site cooperative interactions (Equations 13–14). Figure 5A shows the model predictions in the case where CaM is assumed to have two pairs of independent EF-hand globular domains. Within these domains, one Ca^2+ ^binding site influences the other. In the *N*-terminal domain, the affinity of the second site depends on the state of the first and changes from *K*_2 _= 0.9 to $K_{2}^{coop}$ = 0.2 (*μM l*^-1^) when a Ca^2+ ^ion occupies the first centre. In the *C*-terminal, the affinity of the fourth site depends on the state of the third and changes from *K*_4 _= 0.8 to $K_{4}^{coop}$ = 0.1 (*μM l*^-1^) when a Ca^2+ ^ion occupies the third centre. The model (**Model 2 **in Materials and Methods) predicts that such changes will mainly influence the "amplitudes" of the intermediate conformations of the concentration-dependent profiles while leaving the ligand concentrations that produce their maximum values largely unchanged. Figure 5B shows the difference in the total amount of bound ligand with and without the type of cooperativity described above. Since the amount of bound Ca^2+ ^is frequently measured in Ca^2+^-CaM or other ligand-multisite protein interaction experiments, these results allow a direct quantitative comparison to be made of the binding reactions with and without the presence of cooperative binding.

### *N*- and *C*-terminal domains reveal unique cooperative properties

A more realistic description for EF-hand Ca^2+ ^binding sites would involve the incorporation of the influence of both Ca^2+ ^binding sites on each other within the CaM globular domains as schematically illustrated on Figure 5 (**Model 3 **in Materials and Methods). The resulting system of differential equations describing the Ca^2+^-CaM interactions is given by (Equation 15) in Materials and Methods. The model based on these assumptions leads to some interesting predictions regarding Ca^2+^-dependent CaM interactions with various CaM target protein peptides [33,49] and reveals a complex story of specificity in CaM regulation. While it is well established that Ca^2+ ^ions are required to modulate the CaM-target protein interactions, the mechanism of Ca^2+^-induced CaM conformational transitions that allow selective interactions with a particular target protein is still unclear. The presented model for Ca^2+ ^binding to CaM provides new insights into how the cooperative interactions between EF-hand binding sites contribute to the mechanism of selective target regulation by CaM, as described below.

**Figure 6:**
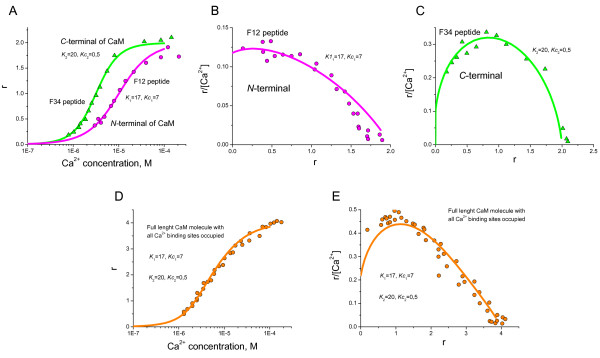
**Analysis of cooperativity in Ca^2+ ^binding to CaM fragments and full CaM molecules**. Ca^2+ ^binding to scallop testis CaM *N*- and *C*-terminal domains (A) as well as full length CaM (D) was measured by flow dialysis in [12]. The Scatchard plots for *N*-terminal (B), *C*-terminal (C) domains, and full length CaM (E) suggest that Ca^2+ ^binding sites are cooperatively bound in pairs within the *N*- and *C*- terminal globular domains. r is the number of mol of bound Ca^2+ ^per mol of CaM [12]. *K*_1 _and *Kc*_1 _are the dissociation and cooperative dissociation constants for the *N*-terminal, while *K*_2 _and *Kc*_2 _are the dissociation and cooperative dissociation constants for the C-terminal of scallop testis CaM.

To analyse the pairwise cooperativity between the EF-hand binding sites of the *N*- and *C*- terminals of CaM, our model has been applied to the experiments on Ca^2+ ^binding to tryptic fragments of CaM. Here, the conditional probabilities for EF-hand Ca^2+ ^binding sites to be free or occupied corresponding to detailed pairwise cooperative mechanisms in the *N*- and *C*-terminal domain of CaM are used. Two dissociation constants are introduced for the EF-hand binding sites, *K*_1 _and *Kc*_1_. The dissociation constant for the binding centres changes from *K*_1 _to *Kc*_1 _when a neighbouring Ca^2+ ^binding loop becomes occupied by a Ca^2+ ^ion in either the *N*- or *C*- terminal domain of the CaM molecule. The comparison of the model predictions with the experimental results (Figure 6A) reveals that the Ca^2+ ^binding sites of both *N*- (Figure 6B) and *C*- terminals (Figure 6C) have similar dissociation constants *K*_1_. However, the *C*- terminal exhibited much higher affinity for a cooperatively influenced binding site (for a Ca^2+ ^binding site when the neighbouring site is occupied). Figure 6D shows the amount of Ca^2+ ^bound to full length CaM while Figure 6E shows the corresponding the Scatchard plot. The dissociation constants shown are the pre- and post-occupied *K*_1 _and *K*_2 _dissociation constants for the Ca^2+ ^binding sites in *N*- and *C*-terminal domains. It, therefore, appears possible that the *N*- and *C*- terminals of CaM can be distinguished based on the unique sets of cooperativity-dependent dissociation constants rather than by the absolute dissociation constants. It is important to notice that this analysis concentrated on differences in the Ca^2+^-CaM dissociation constants and did not consider the differences in dissociation constants between intermediate Ca^2+^-CaM complexes and target proteins. The incorporation of this additional important parameter would serve to further improve the description of the Ca^2+^-CaM-dependent regulation.

### Calmodulin-target interactions reveal target-specific cooperativity in Ca^2+ ^binding

**Figure 7:**
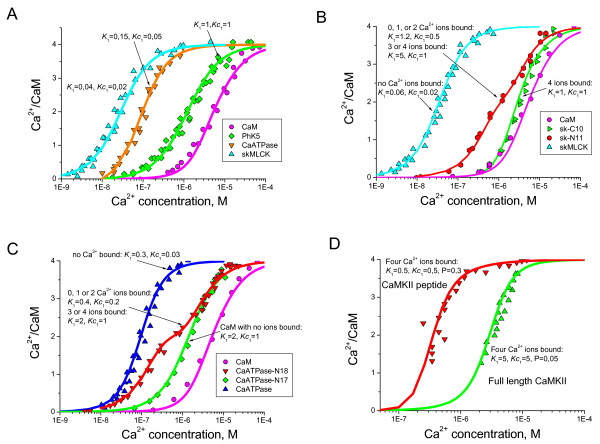
**Ca^2+^-dependent interaction of CaM with target protein peptides**. The data from [33] shows Ca^2+ ^binding to CaM in the presence of peptides derived from phosphorylase kinase (PhK5), erythrocyte Ca^2+ ^CaATPase, skeletal Myosin Light Chain Kinase (skMLCK) (A). (B) and (C) show the Ca^2+ ^binding to CaM in the presence of parts of skMLCK and CaATPase peptides, respectively [33]. Interaction data for Ca^2+^-CaM complexes in the presence and absence of protein kinase II (CaMPKII) reveal parameters of complex formation reaction [49]. The solid line in each case shows the model prediction for Ca^2+^-CaM binding. *K*_1 _is macroscopic Ca^2+ ^dissociation constant, *Kc*_1 _is the cooperatively affected dissociation constants. Arrows indicate the number of Ca^2+ ^ions bound to CaM required to create a complex with a target molecule peptide.

A critical aspect of Ca^2+^-CaM-target complex formation is the order of the assembly and the alterations of macroscopic dissociation constants for Ca^2+ ^at individual sites, depending on whether CaM is bound to the target protein. The current model has been applied to the dose-response curves for Ca^2+^-CaM-target peptide complexes in an attempt to distinguish the following details: i) the sets of cooperative dissociation constants for the *N*- and *C*-terminal domains of CaM, ii) the sequence of Ca^2+^-CaM-target (Ca^2+^-CaM + target or CaM-target + Ca^2+^) complex assembly, iii) the contribution of Ca^2+ ^unsaturated CaM conformation species. To obtain insights into these questions, the model predictions for the dose-response curves of these peptides were compared with the experimentally established Ca^2+ ^concentration-dependent profiles from [33] and [49]. While fitting the model predictions to the experimental data from [33] we assumed that CaM may bind target proteins with different numbers of bound Ca^2+ ^ions or even in the Ca^2+ ^free state. Figure 7 shows the Ca^2+^-dependent profiles of Ca^2+^-CaM and Ca^2+^-CaM in complex with peptides derived from phosphorylase kinase (PhK5), erythrocyte Ca^2+^-ATPase and skeletal myosin light chain kinase (skMLCK) (A), Ca^2+^-CaM bound to half length peptides sk-C10 and sk-N11 of skMLCK (B), Ca^2+^-CaM bound to half length peptides N17 and N18 of CaATPase (C), and CaMKII and CaMPII-cbp peptide (D). The model predictions in comparison with the experimental data suggest that it is possible that all conformations may have their own *K*_1 _and *K*_2 _dissociation constants. The target proteins may interact with a specific Ca^2+^-CaM complex or with a combination of CaM conformations with a unique number of Ca^2+ ^ions. It also appears plausible that Ca^2+ ^binding sites on CaM have different affinities for Ca^2+ ^ions, depending on whether CaM-target or Ca^2+^-CaM binding occurs first, in addition to pairwise cooperativity between Ca^2+ ^binding sites.

The results of the present study, when combined with previous experimental data from the literature, suggest that CaM interacts with phosphorylase kinase, CaATPase and skMLCK in the apo state, but activates these proteins only when Ca^2+ ^ions bind to a CaM-target protein complex. CaMKII kinase, on the other hand, binds to the Ca^2+^-CaM complex rather than apo-CaM. Each kinase has a unique combination of *K*_1 _and *K*_2 _dissociation constants. The half length peptides of CaATPase and skMLCK appear to have an even more complex mechanism of binding. As mentioned earlier, each Ca^2+^-CaM complex (with variable numbers of Ca^2+ ^ions bound) may have a unique set of *K*_1 _and *K*_2 _constants for a target protein or peptide. For simplicity, all possible CaM species were divided into two groups: i) those with less than 3 Ca^2+ ^ions bound and ii) those with three and four Ca^2+ ^ions bound to CaM. A comparison of the model predictions with the Ca^2+^-CaM-parts of skMLCK and CaATPase peptide binding data (Figure 7B and 7C) suggests that both the *K*_1 _and the cooperatively influenced *K*_2 _dissociation constants are different when peptides are bound to CaM species with less than 3 Ca^2+ ^ions or to CaM species with 3 or 4 ions. The conclusion from this observation is that the specificity in Ca^2+^-CaM-dependent regulation arises from a combination of the target specific affinity between Ca^2+ ^and CaM, target specific cooperative constants, the order of the Ca^2+^-CaM-target complex assembly, as well as the number of Ca^2+ ^ions bound to CaM. All these factors contribute to the mechanism of selective Ca^2+^-CaM dependent regulation in addition to the diversity of CaM-target interfaces [39].

### pH dependence of cooperative Ca^2+ ^binding to CaM

**Figure 8:**
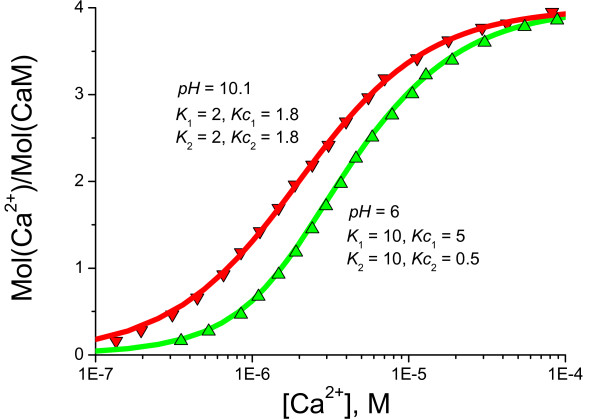
**pH dependence of Ca^2+ ^binding to CaM**. Ca^2+ ^binding to CaM under different pH conditions measured by [9] can be explained in terms of altered dissociation and cooperative dissociation constants. The increase of pH raises affinity to Ca^2+ ^and eliminates cooperativity between Ca^2+ ^binding sites on CaM.

Ca^2+ ^binding to CaM has been reported to be dependent on the acidity of the solution [9]. The detailed CaM model comprising symmetrical pairwise influence of Ca^2+ ^binding sites in *N*- and *C*- terminal domains has been employed to investigate the impact of pH on the cooperative Ca^2+ ^binding. It is established that the macroscopic dissociation constants at the *N*- and *C*- terminal differ by an order of magnitude. The two EF-hand globular domains of CaM appear to play separate roles in intracellular signalling by selectively modulating various effectors [4,49]. The model predictions in comparison with the experimental data for Ca^2+ ^binding to CaM published in [9] reveal that protonation decreases Ca^2+ ^affinity to CaM. This result exemplifies the advantages of the proposed detailed Ca^2+ ^binding model to CaM over the Hill and Adair equations in allowing enhanced interpretation of the same experimental dose-response curves. Interestingly, in this case it suggests the complete elimination of cooperativity between Ca^2+ ^binding sites (Figure 8) in both *N*- and *C*- terminals under increased pH conditions. Finally, Table 1 compares the dissociation constants derived in those studies cited above where the experimental results were fitted by the Hill and Adair equations, with those produced by the proposed model. The significant differences in the values of the resulting dissociation constants reflect the different assumptions made about the mechanism of Ca^2+^-CaM interactions, and highlight the importance of developing detailed mathematical models which specifically reflect the structure of the molecular interactions being considered.

**Table 1:** Ca^2+^-CaM dissociation constants derived by the different mathematical models.

Proteins	Hill	Adair	present model	Reference
PhK5	*K*_*D*1 _= 0.24, *K*_*D*2 _= 13		*K*_1 _= 1, *Kc*_1 _= 1, *K*_2 _= 1, *Kc*_2 _= 1	[33]
skMLCK	*K*_*D*1 _= 0.02, *K*_*D*2 _= 0.08		*K*_1 _= 0.04, *Kc*_1 _= 0.02, *K*_2 _= 0.04, *Kc*_2 _= 0.02	
sk-N11	*K*_*D*1 _= 0.26, *K*_*D*2 _= 6		*K*_1 _= 1.2, *K*_2 _= 0.5 *K*_1 _= 5, *K*_2 _= 1	
sk-C10	*K*_*D*1 _= 3.4, *K*_*D*2 _= 4		*K*_1 _= 0.06, *Kc*_1 _= 0.02, *K*_2 _= 0.06, *Kc*_2 _= 0.02	
CaATPase	*K*_*D*1 _= 0.09, *K*_*D*2 _= 0.2		*K*_1 _= 0.15, *Kc*_1 _= 0.05, *K*_2 _= 0.15, *Kc*_2 _= 0.05	
ATPase-N18	*K*_*D*1 _= 0.12, *K*_*D*2 _= 3.9		*K*_1 _= 2, *Kc*_1 _= 1, *K*_2 _= 2, *Kc*_2 _= 1	
ATPase-C17	*K*_*D*1 _= 0.66, *K*_*D*2 _= 2.4		*K*_1 _= 0.4, *Kc*_1 _= 0.2 *K*_2 _= 2, *Kc*_2 _= 1	
CaMKII-cbp			*K*_1 _= 0.5, *Kc*_1 _= 0.5, *K*_2 _= 0.5, *Kc*_2 _= 0.5	[49]
CaMKII			*K*_1 _= 5, *Kc*_1 _= 5, *K*_2 _= 5, *Kc*_2 _= 5	
CaM pH = 7.2		*K*_1 _= 0.34, *K*_2 _= 0.36, *K*_3 _= 0.13, *K*_4 _= 0.06	*K*_1 _= 17, *Kc*_1 _= 7, *K*_2 _= 20, *Kc*_2 _= 0.5	[12]
F12		*K*_1 _= 0.142, *K*_2 _= 0.062	*K*_1 _= 17, *Kc*_1 _= 7, *K*_2 _= 17, *Kc*_2 _= 7	
F34		*K*_3 _= 0.0543, *K*_4 _= 1.82	*K*_1 _= 20, *Kc*_1 _= 0.5, *K*_2 _= 20, *Kc*_2 _= 0.5	
CaM pH = 6			*K*_1 _= 10, *Kc*_1 _= 5, *K*_2 _= 10, *Kc*_2 _= 5	[9]
CaM pH = 10.1			*K*_1 _= 2, *Kc*_1 _= 1.8, *K*_2 _= 2, *Kc*_2 _= 1.8	

*K*_1 _is the dissociation constant for a Ca^2+ ^binding site in the *N*-terminal, *Kc*_1 _is a cooperatively modified dissociation constant for a Ca^2+ ^binding site in the *N*-terminal when a neighbouring site is occupied, *K*_2 _is a dissociation constant for a Ca^2+ ^binding site in the *C*-terminal, and *Kc*_2 _is a cooperatively influenced dissociation constant for a Ca^2+ ^binding site in the *C*-terminal when a neighbouring site is occupied. All constants shown are in μM.

## Discussion

In this paper a structural model of CaM interactions with and without cooperativity has been used to elucidate the mechanisms of Ca^2+^-CaM-target complex assembly. The differences seen in dose-response curves for proteins activated by Ca^2+^-CaM pairs were explained in terms of cooperative interactions between the EF-hand pairs [38,40,41] in both CaM domains. This study predicts that the specific interaction interface between CaM and CaM-regulated proteins [8,10,39] is complemented by a number of additional factors influencing the Ca^2+^-CaM-target complex assembly. By comparing our model predictions with experimentally measured dose-response curves from the literature, we propose that some proteins bind CaM without Ca^2+ ^ions and only become activated when Ca^2+ ^ions interact with the CaM-target complex, whereas others are activated by CaM molecules with already bound Ca^2+ ^ions. The Ca^2+^-CaM interaction properties are tuned by the target proteins and characterized not only by the macroscopic dissociation constant set for Ca^2+ ^sites, but also by the macroscopic cooperatively altered dissociation constants that are also unique to the CaM binding proteins. In other words, the order of Ca^2+^-CaM-target complex assembly, the number of bound Ca^2+ ^ions, target specific Ca^2+^-CaM cooperative affinities, in addition to unique CaM-target interaction interfaces, all allow CaM to achieve its highly versatile intracellular multifunctionality. This proposition also explains the effects of pH on the considered dose-response curves by allowing for the modulation of the cooperatively effected dissociation constants. We would also like to point out that while we addressed the Ca^2+^-CaM interactions in great detail, the model could still be developed further by incorporating detailed dissociation constants between the intermediate Ca^2+^-CaM complexes and target proteins in a similar way to how it has been done for the Ca^2+^-CaM interactions.

Although the presented model predicts similar curves to the ones already used to approximate the experimental Ca^2+^-CaM-target dose-response data using the Hill and Adair models, it allows a far more detailed interpretation of the Ca^2+^-CaM dependent interactions involved. In particular, it distinguishes the structure-dependent properties of CaM molecules and suggests potential scenarios for Ca^2+^-CaM-target complex assembly. Importantly, it reveals the CaM specific type of cooperativity involved in this process and helps to explain the contribution of cooperativity in the specificity of CaM-dependent regulation.

## Conclusion

We propose a number of conclusions from this study which, we believe, contribute to improving our understanding of intracellular CaM regulation and provide useful hypotheses for further experimental validation.

(1) Mathematical models for protein interactions are usually derived according to a number of assumptions which will inevitably be more or less applicable to each particular protein. The structure of CaM suggests that this molecule is very likely to have non-sequential Ca^2+ ^access to EF-hand binding sites. The results of our analysis support the theory of non-sequential cooperative access of Ca^2+ ^to CaM binding sites, and also allow the derivation of cooperatively effected dissociation constants, thus providing a more realistic tool for fitting experimental dose-response curves.

(2) Our model suggests that the structural data alone cannot provide the required level of information and comparisons with the dose-response data are required. Predictions from the mathematical model used in this study were compared with the dose-response curves for Ca^2+ ^binding to CaM and Ca^2+^-CaM-target peptides. This analysis allowed us to distinguish between (a) proteins that form a complex with CaM in its Ca^2+ ^free state and then interact with Ca^2+ ^ions and (b) other proteins which interact with Ca^2+ ^bound CaM with variable numbers of Ca^2+ ^ions. However, the transient kinetics has not been addressed in this study.

(3) In addition to the diversity of interaction interfaces, the specificity and selectivity of CaM target activation may be achieved by variations in the target-specific dissociation and cooperatively effected dissociation constants, the order of Ca^2+^-CaM-target complex assembly and the number of Ca^2+ ^ions required to bind CaM for target activation.

## Methods

### A structural mathematical model for Ca^2+^-CaM activation

Here, we present the mathematical equations used to describe Ca^2+^-CaM interactions in our study. To clarify how various factors contribute to the CaM-dependent regulation, we describe three models for CaM starting from a very basic approximation of completely independent Ca^2+ ^binding sites and gradually progressing to more realistic models that take account of cooperativity mechanisms and the Ca^2+^-CaM-target peptide complex assembly.

### Model 1. CaM with independent Ca^2+ ^binding sites

In this model we assume that at very basic level of Ca^2+^-CaM interactions, Ca^2+ ^binding sites can be considered independent. We therefore derive the equations for the case of independent binding. We assume that CaM undergoes a conformational transition upon Ca^2+ ^binding and adopts a unique conformation according to the number of bound ions. We will denote CaM conformations by *cm*_*j*_, *j *= 0,1, ..., 4. *cm*_0 _is a conformation with no bound ligand molecules and *cm*_1 _is a conformation with one bound ligand molecule. The concentration of CaM conformation with a given number of bound ligand molecules as a function of ligand concentration is given by:

(1) $$cm_{i}(u)=cm0\cdot \prod_{i=1}^{n}p_{i}^{c_{i}}(u).$$

where *cm*0 is the total concentration of CaM, *u *is normalised Ca^2+ ^concentration,$p_{i}^{0}(u)$ is the probability of binding site *i *not being occupied and $p_{i}^{1}(u)$ is the probability of binding site *i *being bound. *c*_*i *_equals 1 if a binding site is occupied and 0 if it is not. The probability of CaM being in a particular bound state is equal to the product of the probabilities of each individual binding site.

The probabilities for a binding site to be not occupied or occupied as a function of Ca^2+ ^concentration are given by:

(2) $$\begin{array}{l} p^{0}(u)=\frac{K}{K+u},\\ p^{1}(u)=\frac{u}{K+u}. \end{array}$$

where the *K *and *u *are the microscopic equilibrium dissociation constant and the ligand concentration, respectively. Effectively these are Michaelis-Menten equations for a protein in a complex with and without a ligand molecule, but normalized by the total protein concentration.

The multiplication of probabilities from (2) for occupied sites gives an equation for a fully bound protein with *n *binding sites:

(3) $$p(u)=\frac{u^{n}}{{\left(K+u\right)}^{n}},$$

where the *K *and *u *are the equilibrium dissociation constant and the ligand concentration, respectively.

Other well known models to describe Ca^2+ ^binding to CaM are the Hill [42] and the modified Adair [11,52] equations:

(4) $$\begin{array}{l} p_{Hill}(u)=\frac{u^{n}}{K^{n}+u^{n}},\\ p_{Adair}(u)=\frac{u^{n}}{\sum_{i=0}^{n}u^{i}\cdot K^{n-i}}, \end{array}$$

A complete mathematical description of the relationship between macroscopic constants derived from the Adair equation and the proposed model is provided in the Supplementary Materials Section (Additional File 1).

In the most general case, the concentration of any multisite protein *L*_*i *_(*u*) with *n *ligand binding sites in a particular ligand-bound state is given by the multiplication of probabilities (2):

(5) $$L_{i}(u)=L0\cdot \frac{u^{i}\cdot \prod_{j=n-i}^{n}K_{j}}{\prod_{j=1}^{n}\left(K_{j}+u\right)}$$

where *L*0 is the total protein concentration and *L*_*i *_is the concentration of conformation *i*, *K*_*j *_are the equilibrium dissociation constants of each binding site, and *u *is the ligand concentration.

If a protein has identical binding sites, equation 5 simplifies to the following formula:

(6) $$L_{i}(u)=L0\cdot \frac{u^{i}\cdot K^{n-i}}{{\left(K+u\right)}^{n}},i=0,1,...,n.$$

The conformations *L*_1_(*u*), ..., *L*_*n*-1_(*u*) of a multisite protein are all bell-shaped curves, the conformation *L*_0_(*u*) is the apo state of a multisite protein, whereas the *L*_*n*_(*u*) is the fully bound multisite protein. If equation (5) is divided by *L*0, then instead of predicting protein concentrations in specific ligand-bound conformations, it predicts the probability of a particular conformation to be in that state as a function of ligand concentration.

The formula predicting the concentration of CaM in complex with target protein *N *as a function of Ca^2+ ^concentration is given by:

(7) $$cm_{i}N(cm_{i}(u))=N0\cdot \frac{cm_{i}(u)}{K_{d}+cm_{i}(u)},$$

where *cm*_*i*_(*u*) is substituted from equation 5, and *K*_d _is the dissociation constant for CaM-target interactions, *N*0 is the total concentration of target protein.

### Model 2. Cooperative Ca^2+^-CaM interactions

While the previous model provides predictions for the number of Ca^2+^-CaM complexes as a function of Ca^2+ ^concentration with a reasonable accuracy, it does not capture the effects of the cooperative influence of Ca^2+ ^binding sites. There are several possible ways to incorporate these cooperative mechanisms into the model. In order to derive a model that illustrates what contribution cooperativity makes to the distribution of concentration profiles of Ca^2+^-CaM complexes, we assume that in the *N*-terminal domain, the first centre is cooperatively bound to the second, and in the *C*-terminal, the third is cooperatively bound to the fourth. In this case, we will define the dissociation constants as *K*_1_, *K*_2_, $K_{2}^{coop}$, *K*_3_, *K*_4_, $K_{4}^{coop}$, where $K_{2}^{coop}$ and $K_{4}^{coop}$ are the cooperatively influenced dissociation constants for the second and fourth centres when the ligand is bound to the first and third binding sites, correspondingly.

The probabilities for the first binding site to be free $p_{1}^{0}$ or occupied $p_{1}^{1}$ are given by:

(8) $$\begin{array}{l} p_{1}^{0}=\frac{K_{1}}{K_{1}+u},\\ p_{1}^{1}=\frac{u}{K_{1}+u}. \end{array}$$

where *K*_1 _and *u *are the equilibrium dissociation constant for the first centre and the ligand concentration, respectively.

The probabilities for the second centre to be in a particular state are:

(9) $$\begin{array}{l} p_{2}^{00}=\frac{K_{1}}{K_{1}+u}\cdot \frac{K_{2}}{K_{2}+u},\\ p_{2}^{10}=\frac{u}{K_{1}+u}\cdot \frac{K_{2}^{coop}}{K_{2}^{coop}+u},\\ p_{2}^{01}=\frac{K_{1}}{K_{1}+u}\cdot \frac{u}{K_{2}+u},\\ p_{2}^{11}=\frac{u}{K_{1}+u}\cdot \frac{u}{K_{2}^{coop}+u}. \end{array}$$

where $p_{2}^{00}$ is the probability for both the first and the second centres to be free, $p_{2}^{01}$ is the probability for the first site to be free and the second to be occupied, $p_{2}^{10}$ is the probability for the first site to be bound and the second to be free, and $p_{2}^{11}$ is the probability for both sites to be ligand bound. *K*_2 _and $K_{2}^{coop}$ are the dissociation and the cooperatively modified dissociation constants for the second Ca^2+ ^binding site.

The probabilities for the third centre are:

(10) $$\begin{array}{l} p_{3}^{0}=\frac{K_{3}}{K_{3}+u},\\ p_{3}^{1}=\frac{u}{K_{3}+u}. \end{array}$$

where *K*_3 _is a dissociation of the third binding site.

The probabilities for the fourth site are given by:

(11) $$\begin{array}{l} p_{4}^{00}=\frac{K_{3}}{K_{3}+u}\cdot \frac{K_{4}}{K_{4}+u},\\ p_{4}^{10}=\frac{u}{K_{3}+u}\cdot \frac{K_{4}^{coop}}{K_{4}^{coop}+u},\\ p_{4}^{01}=\frac{K_{3}}{K_{3}+u}\cdot \frac{u}{K_{4}+u},\\ p_{4}^{11}=\frac{u}{K_{3}+u}\cdot \frac{u}{K_{4}^{coop}+u}. \end{array}$$

where $p_{4}^{00}$ is the probability for both the third and the fourth centres to be free, $p_{4}^{01}$ is the probability for the third site to be free and the fourth to be occupied, $p_{3}^{10}$ is the probability for the third site to be bound and the fourth to be free, and $p_{4}^{11}$ is the probability for both sites to be ligand bound. *K*_4 _and $K_{4}^{coop}$ are the dissociation and the cooperatively modified dissociation constants for the second Ca^2+ ^binding site.

The probabilities of *cm*_i _conformations to be in a particular conformation with i bound ligand molecules as a function of ligand concentration are:

(12) $$cm_{i}=p_{2}^{k_{1},k_{2}}\cdot p_{4}^{k_{3},k_{4}},$$

where *k*_i _= 0, if the binding site *i *is not occupied and *k*_i _= 1 if the centre *i *is occupied by a ligand molecule.

The distribution of intermediate Ca^2+^-CaM complexes with 1, 2 and 3 Ca^2+ ^ions with and without cooperativity is shown in Figure 5A.

We next compare the amount of bound ligand in the presence and in the absence of the cooperative mechanism. In the absence of any cooperativity the multisite protein binds ligand molecules according to the equation:

(13) $$Ls(u)=L0\cdot \sum_{i=1}^{n}\frac{u}{K_{i}+u}.$$

In the case of pairs of cooperatively interacting centres, the amount of bound ligand is given by:

(14) $$Ls^{coop}(u)=L0\cdot \left(\frac{u^{2}\cdot (K_{2}-K_{2}^{coop})}{\left(K_{1}+u)\cdot (K_{2}+u)\cdot (K_{2}^{coop}+u\right)}+\frac{u^{2}\cdot (K_{4}-K_{4}^{coop})}{\left(K_{3}+u)\cdot (K_{4}+u)\cdot (K_{4}^{coop}+u\right)}+\sum_{i=1}^{n}\frac{u}{K_{i}+u}\right).$$

Figure 5B shows the total amount of ligand bound to CaM in the presence (1) and absence (2) of cooperative binding. The line (3) shows the difference in the level of bound ligand between the two types of binding mechanisms.

### Model 3. The Ca^2+^-CaM-target protein complex assembly

In the previous model we have assumed that the second binding site was cooperatively dependent on the first, but the first site was not dependent on the second. Similar assumptions were made for the *C*-terminal domain. A more precise description would also assume the first Ca^2+ ^binding site cooperatively depends on the second and the third Ca^2+ ^binding site depends on the fourth binding sites. For two Ca^2+ ^binding sites in the *N*-terminal domain, the more realistic case is described by the following system of differential equations:

(15) $$\begin{array}{l} \frac{d}{dt}cm_{00}=-k_{1}\cdot cm_{00}\cdot u-k_{2}\cdot cm_{00}\cdot u+d_{1}\cdot cm_{10}+d_{2}\cdot cm_{01}\\ \frac{d}{dt}cm_{10}=k_{1}\cdot cm_{00}\cdot u-d_{1}\cdot cm_{10}-kc_{2}\cdot cm_{10}\cdot u+dc_{2}\cdot cm_{11}\\ \frac{d}{dt}cm_{01}=k_{2}\cdot cm_{00}\cdot u-d_{2}\cdot cm_{01}-kc_{1}\cdot cm_{01}\cdot u+dc_{1}\cdot cm_{11}\\ \frac{d}{dt}cm_{11}=-kc_{1}\cdot cm_{01}\cdot u-kc_{2}\cdot cm_{10}\cdot u+dc_{1}\cdot cm_{11}+dc_{2}\cdot cm_{11} \end{array},$$

where *cm*_00_, *cm*_01_, *cm*_10_, *cm*_11 _are CaM molecules without Ca^2+ ^ions, with one Ca^2+ ^ion bound to the *N*-terminal domain, with one Ca^2+ ^ion bound to the *N*-terminal domain, and CaM species with two bound Ca^2+ ^ions at each terminal domain, respectively. *k*_1 _and *k*_2 _are the association constants and *kc*_1 _and *kc*_2 _are the cooperatively modified association constants for the *N*- terminal binding sites of CaM, respectively. Similarly, *d*_1_, *d*_2_, *dc*_1_, *dc*_2 _are the dissociation and cooperatively modified dissociation constants for the *N*- and terminal binding sites, respectively. Note that a similar system of differential equations can be developed for the *C*-terminal.

The conservation law gives:

(16) *cm*_00 _+*cm*_10 _+ *cm*_01 _+ *cm*_11 _= 1

In steady-state, the matrix for the system (15) with the last equation substituted by (16) is given by:

(17) $$N=\left(\begin{array}{cccc} -(k_{1}+k_{2})\cdot u & d_{1} & d_{2} & 0\\ k_{1}\cdot u & -d_{1}-kc_{2}\cdot u & 0 & dc_{2}\\ k_{2}\cdot u & 0 & -d_{2}-kc_{1}\cdot u & dc_{1}\\ 1 & 1 & 1 & 1 \end{array}\right)$$

The matrix (17) can be solved using Cramer's method. The determinant of the matrix is given by:

(18) $$Det_{N}(u)=u^{3}+K_{1}\cdot (2\cdot Kc_{1}+1)\cdot u^{2}+3\cdot K_{1}\cdot Kc_{1}\cdot u+K_{1}^{2}\cdot Kc_{1}$$

where $K_{1}=\frac{d_{1}}{k_{1}}$, $Kc_{1}=\frac{dc_{1}}{kc}$. *k*_1 _= *k*_2 _= *kc*_1 _= *kc*_2 _is a simplifying assumption that allows analytical solution of the current system.

The matrices for the individual species of the *N*-terminal domain are given by:

(19) $$N_{00}=\left(\begin{array}{cccc} 0 & d_{1} & d_{2} & 0\\ 0 & -d_{1}-kc_{2}\cdot u & 0 & dc_{2}\\ 0 & 0 & -d_{2}-kc_{1}\cdot u & dc_{1}\\ 1 & 1 & 1 & 1 \end{array}\right)$$

(20) $$N_{01}=\left(\begin{array}{cccc} -u\cdot (k_{1}+k_{2}) & d_{1} & 0 & 0\\ k_{1}\cdot u & -d_{1}-kc_{2}\cdot u & 0 & dc_{2}\\ k_{2}\cdot u & 0 & 0 & dc_{1}\\ 1 & 1 & 1 & 1 \end{array}\right)$$

(21) $$N_{10}=\left(\begin{array}{cccc} -u\cdot (k_{1}+k_{2}) & 0 & d_{2} & 0\\ k_{1}\cdot u & 0 & 0 & dc_{2}\\ k_{2}\cdot u & 0 & -d_{2}-kc_{1}\cdot u & dc_{1}\\ 1 & 1 & 1 & 1 \end{array}\right)$$

(22) $$N_{11}=\left(\begin{array}{cccc} u\cdot (k_{1}+k_{2}) & d_{1} & d_{2} & 0\\ k_{1}\cdot u & -d_{1}-kc_{2}\cdot u & 0 & 0\\ k_{2}\cdot u & 0 & -d_{2}-kc_{1}\cdot u & 0\\ 1 & 1 & 1 & 1 \end{array}\right)$$

where *N*_00_, *N*_01_, *N*_10 _and *N*_11 _correspond to the *N*-terminal domain without any Ca^2+ ^ions bound, with one Ca^2+ ^ion bound to one or another binding site and to the fully bound state, respectively.

The determinants of the matrices (19–22) are given by:

(23) $$\begin{array}{l} Det_{N_{00}}(u)=K_{1}\cdot Kc_{1}\cdot (K_{1}+u),\\ Det_{N_{01}}(u)=Kc_{1}\cdot u\cdot (K_{1}+u),\\ Det_{N_{10}}(u)=Kc_{1}\cdot u\cdot (K_{1}+u),\\ Det_{N_{11}}(u)=K_{1}\cdot u^{2}\cdot (1+u). \end{array}$$

The probabilities for the *N*-terminal to be in a particular state with variable numbers of Ca^2+ ^ions are given by:

(24) $$\begin{array}{l} p_{N_{00}}(u)=\frac{Det_{N_{00}}(u)}{Det_{N}(u)},\\ p_{N_{01}}(u)=\frac{Det_{N_{01}}(u)}{Det_{N}(u)},\\ p_{N_{10}}(u)=\frac{Det_{N_{10}}(u)}{Det_{N}(u)},\\ p_{N_{11}}(u)=\frac{Det_{N_{11}}(u)}{Det_{N}(u)} \end{array}$$

where $p_{N_{00}}(u)$ is the probability for the *N*-terminal to be in a Ca^2+ ^state, $p_{N_{01}}(u)$ and $p_{N_{10}}(u)$ describe the probabilities for a complex with one Ca^2+ ^ion, and $p_{N_{11}}(u)$ is the probability function for the *N*-terminal to be occupied by Ca^2+^.

In order to derive the steady-state dependence of the *C*-terminal state as a function of Ca^2+ ^concentration, a similar procedure can be applied. Similarly to (17) and (18), the determinant for the *C*-terminal is given by:

(25) $$Det_{C}(u)=u^{3}+K_{2}\cdot (2\cdot Kc_{2}+1)\cdot u^{2}+3\cdot K_{2}\cdot Kc_{2}\cdot u+K_{2}^{2}\cdot Kc_{2}$$

Where $K_{2}=\frac{d_{2}}{k_{2}},Kc_{2}=\frac{dc_{2}}{kc_{2}},k_{2}=kc_{2}$

Similarly to (19–23), the solution for the *C*-terminal states is given by:

(26) $$\begin{array}{l} Det_{C_{00}}(u)=K_{2}\cdot Kc_{2}\cdot (K_{2}+u),\\ Det_{C_{01}}(u)=Kc_{2}\cdot u\cdot (K_{2}+u),\\ Det_{C_{10}}(u)=Kc_{2}\cdot u\cdot (K_{2}+u),\\ Det_{C_{11}}(u)=K_{2}\cdot u^{2}\cdot (1+u). \end{array}$$

The probabilities for the *C*-terminal to be in a particular state with variable number of Ca^2+ ^ions are given by:

(27) $$\begin{array}{l} p_{C_{00}}(u)=\frac{Det_{C_{00}}(u)}{Det_{C}(u)},\\ p_{C_{01}}(u)=\frac{Det_{C_{01}}(u)}{Det_{C}(u)},\\ p_{C_{10}}(u)=\frac{Det_{C_{10}}(u)}{Det_{C}(u)},\\ p_{C_{11}}(u)=\frac{Det_{C_{11}}(u)}{Det_{C}(u)} \end{array}$$

where $p_{N_{00}}(u)$ is the probability for the *N*-terminal to be in a Ca^2+ ^state, $p_{N_{01}}(u)$ and $p_{N_{10}}(u)$ describe the probabilities for a complex with one Ca^2+ ^ion, and $p_{N_{11}}(u)$ is the probability function for the *N*-terminal occupied by Ca^2+^.

The combination of (24) and (27) provides the solution for individual CaM species with variable numbers of Ca^2+ ^ions:

(28) $$\begin{array}{l} CaM_{00}^{00}=CaM0\cdot \frac{Det_{N_{00}}(u)\cdot Det_{C_{00}}(u)}{Det_{N}(u)\cdot Det_{C}(u)},\\ CaM_{00}^{01}=CaM_{00}^{10}=CaM0\cdot \frac{Det_{N_{01}}(u)\cdot Det_{C_{00}}(u)}{Det_{N}(u)\cdot Det_{C}(u)},\\ CaM_{00}^{11}=CaM0\cdot \frac{Det_{N_{11}}(u)\cdot Det_{C_{00}}(u)}{Det_{N}(u)\cdot Det_{C}(u)},\\ CaM_{01}^{00}=CaM_{10}^{00}=CaM0\cdot \frac{Det_{N_{00}}(u)\cdot Det_{C_{01}}(u)}{Det_{N}(u)\cdot Det_{C}(u)},\\ CaM_{11}^{00}=CaM0\cdot \frac{Det_{N_{00}}(u)\cdot Det_{C_{11}}(u)}{Det_{N}(u)\cdot Det_{C}(u)},\\ CaM_{11}^{11}=CaM0\cdot \frac{Det_{N_{11}}(u)\cdot Det_{C_{11}}(u)}{Det_{N}(u)\cdot Det_{C}(u)}. \end{array}$$

where $CaM_{00}^{00}$ are CaM species in apo state, $CaM_{11}^{11}$ is fully bound CaM, and $CaM_{00}^{11}$ and $CaM_{11}^{00}$ are CaM species with fully bound *N*- and *C*-terminals, respectively. $CaM_{00}^{01}$ and $CaM_{01}^{00}$ are CaM species with one Ca^2+ ^ion bound to *N*- and *C*-terminals, respectively. Comparison of numerical solutions of this system with the available experimental data [9,15,33] allows us to propose that CaM molecules can be represented as a pair of two independent *N*- and *C*-terminal globular domains, each containing two symmetrical and cooperatively bound EF-hand Ca^2+ ^binding sites.

The equations (28) are specific to Ca^2+^-CaM interactions and incorporate the pairwise cooperative interactions between the EF-hand binding sites within the *N*- and *C*-terminals, whereas the *N*- and *C*-terminal domains are considered to be independent of each other. Note that the equations developed here for the Ca^2+^-CaM complexes are essentially different from the Michelis-Menten, Hill and Adair models, and also differ from models with independent binding sites (5) or with limited amounts of cooperativity (11) and (12).

The equations for the Ca^2+^-CaM complexes (28) have been further applied to calculate binding with the target peptides and proteins:

(29) $$Ca^{2+}\text{-}CaM\text{-}target=T0\cdot \frac{\sum CaM_{m,l}^{i,j}(u)}{K_{d}+\sum CaM_{m,l}^{i,j}(u)}$$

where *T*0 is the total concentration of a target protein or a peptide, *K*_*d *_is the equilibrium dissociation constant between CaM and a target protein, and $\sum CaM_{m,l}^{i,j}(u)$ is a single Ca^2+ ^complex or the sum of several CaM complexes.

The combinations of Ca^2+^-CaM complexes have been varied simultaneously with the dissociation constants to fit the experimental data. This analysis allows us to predict the Ca^2+^-CaM complexes required for activation of specific protein targets. The fitting of dissociation constants of Ca^2+ ^binding sites on CaM molecules to the experimental dose-response curves reveals the impact of the target protein on the Ca^2+^-CaM interactions. The dissociation constants calculated based on the cooperative **Model 3, **which also takes into account the impact of target proteins, are compared with the dissociation constants calculated using the Hill and modified Adair equation in the original experimental publications for the same data in Table 1.

## Authors' contributions

NVV developed and implemented the project under the supervision of DGB and NVK. All authors contributed to the analysis of the model, and PH–H provided biological interpretations of the results. All authors contributed to the writing of the final manuscript.

## Supplementary Material

Additional file 1 The relationship between the Adair and independent binding models. The relationship between macroscopic constants derived from the Adair equation and the proposed Ca^2+^-CaM binding model with free Ca^2+ ^access. Click here for file

## References

[B1] Yap KL, Ames JB, Swindells MB, Ikura M (1999). Diversity of conformational states and changes within the EF-hand protein superfamily. Proteins.

[B2] Berridge MJ, Bootman MD, Lipp P (1998). Calcium–a life and death signal. Nature.

[B3] Guo Q, Shen Y, Lee YS, Gibbs CS, Mrksich M, Tang WJ (2005). Structural basis for the interaction of Bordetella pertussis adenylyl cyclase toxin with calmodulin. Embo J.

[B4] Grabarek Z (2005). Structure of a trapped intermediate of calmodulin: calcium regulation of EF-hand proteins from a new perspective. J Mol Biol.

[B5] Elshorst B, Hennig M, Forsterling H, Diener A, Maurer M, Schulte P, Schwalbe H, Griesinger C, Krebs J, Schmid H (1999). NMR solution structure of a complex of calmodulin with a binding peptide of the Ca2+ pump. Biochemistry.

[B6] Drum CL, Yan SZ, Bard J, Shen YQ, Lu D, Soelaiman S, Grabarek Z, Bohm A, Tang WJ (2002). Structural basis for the activation of anthrax adenylyl cyclase exotoxin by calmodulin. Nature.

[B7] Haiech J, Klee CB, Demaille JG (1981). Effects of cations on affinity of calmodulin for calcium: ordered binding of calcium ions allows the specific activation of calmodulin-stimulated enzymes. Biochemistry.

[B8] Hoeflich KP, Ikura M (2002). Calmodulin in action: diversity in target recognition and activation mechanisms. Cell.

[B9] Iida S, Potter JD (1986). Calcium binding to calmodulin. Cooperativity of the calcium-binding sites. J Biochem.

[B10] Ikura M (1996). Calcium binding and conformational response in EF-hand proteins. Trends Biochem Sci.

[B11] Mirzoeva S, Weigand S, Lukas TJ, Shuvalova L, Anderson WF, Watterson DM (1999). Analysis of the functional coupling between Calmodulin's calcium binding and peptide recognition properties. Biochemistry.

[B12] Minowa O, Yagi K (1984). Calcium binding to tryptic fragments of calmodulin. J Biochem.

[B13] Meador WE, Means AR, Quiocho FA (1992). Target enzyme recognition by calmodulin: 2.4 A structure of a calmodulin-peptide complex. Science.

[B14] Maune JF, Klee CB, Beckingham K (1992). Ca2+ binding and conformational change in two series of point mutations to the individual Ca(2+)-binding sites of calmodulin. J Biol Chem.

[B15] Ogawa Y, Tanokura M (1984). Calcium binding to calmodulin: effects of ionic strength, Mg2+, pH and temperature. J Biochem.

[B16] Andre I, Kesvatera T, Jonsson B, Akerfeldt KS, Linse S (2004). The role of electrostatic interactions in calmodulin-peptide complex formation. Biophys J.

[B17] Andre I, Kesvatera T, Jonsson B, Linse S (2006). Salt enhances calmodulin-target interaction. Biophys J.

[B18] Zhang M, Tanaka T, Ikura M (1995). Calcium-induced conformational transition revealed by the solution structure of apo calmodulin. Nat Struct Biol.

[B19] Chattopadhyaya R, Meador WE, Means AR, Quiocho FA (1992). Calmodulin structure refined at 1.7 A resolution. J Mol Biol.

[B20] Ohki S, Iwamoto U, Aimoto S, Yazawa M, Hikichi K (1993). Mg2+ inhibits formation of 4Ca(2+)-calmodulin-enzyme complex at lower Ca2+ concentration. 1H and 113Cd NMR studies. J Biol Chem.

[B21] Ohki S, Ikura M, Zhang M (1997). Identification of Mg2+-binding sites and the role of Mg2+ on target recognition by calmodulin. Biochemistry.

[B22] Osawa M, Tokumitsu H, Swindells MB, Kurihara H, Orita M, Shibanuma T, Furuya T, Ikura M (1999). A novel target recognition revealed by calmodulin in complex with Ca2+-calmodulin-dependent kinase kinase. Nat Struct Biol.

[B23] Matsubara M, Nakatsu T, Kato H, Taniguchi H (2004). Crystal structure of a myristoylated CAP-23/NAP-22 N-terminal domain complexed with Ca2+/calmodulin. Embo J.

[B24] Houdusse A, Gaucher JF, Krementsova E, Mui S, Trybus KM, Cohen C (2006). Crystal structure of apo-calmodulin bound to the first two IQ motifs of myosin V reveals essential recognition features. Proc Natl Acad Sci USA.

[B25] Bahler M, Rhoads A (2002). Calmodulin signaling via the IQ motif. FEBS Lett.

[B26] Schumacher MA, Crum M, Miller MC (2004). Crystal structures of apocalmodulin and an apocalmodulin/SK potassium channel gating domain complex. Structure.

[B27] Schumacher MA, Rivard AF, Bachinger HP, Adelman JP (2001). Structure of the gating domain of a Ca2+-activated K+ channel complexed with Ca2+/calmodulin. Nature.

[B28] Yuan T, Vogel HJ (1999). Substitution of the methionine residues of calmodulin with the unnatural amino acid analogs ethionine and norleucine: biochemical and spectroscopic studies. Protein Sci.

[B29] Corneliussen B, Holm M, Waltersson Y, Onions J, Hallberg B, Thornell A, Grundstrom T (1994). Calcium/calmodulin inhibition of basic-helix-loop-helix transcription factor domains. Nature.

[B30] Larsson G, Schleucher J, Onions J, Hermann S, Grundstrom T, Wijmenga SS (2001). A novel target recognition revealed by calmodulin in complex with the basic helix–loop–helix transcription factor SEF2-1/E2-2. Protein Sci.

[B31] Bayley PM, Findlay WA, Martin SR (1996). Target recognition by calmodulin: dissecting the kinetics and affinity of interaction using short peptide sequences. Protein Sci.

[B32] Green DF, Dennis AT, Fam PS, Tidor B, Jasanoff A (2006). Rational design of new binding specificity by simultaneous mutagenesis of calmodulin and a target peptide. Biochemistry.

[B33] Peersen OB, Madsen TS, Falke JJ (1997). Intermolecular tuning of calmodulin by target peptides and proteins: differential effects on Ca2+ binding and implications for kinase activation. Protein Sci.

[B34] Evenas J, Malmendal A, Thulin E, Carlstrom G, Forsen S (1998). Ca2+ binding and conformational changes in a calmodulin domain. Biochemistry.

[B35] Linse S, Helmersson A, Forsen S (1991). Calcium binding to calmodulin and its globular domains. J Biol Chem.

[B36] Tan RY, Mabuchi Y, Grabarek Z (1996). Blocking the Ca2+-induced conformational transitions in calmodulin with disulfide bonds. J Biol Chem.

[B37] Wang CL (1985). A note on Ca2+ binding to calmodulin. Biochem Biophys Res Commun.

[B38] Gifford JL, Walsh MP, Vogel HJ (2007). Structures and metal-ion-binding properties of the Ca2+-binding helix-loop-helix EF-hand motifs. Biochem J.

[B39] Bhattacharya S, Bunick CG, Chazin WJ (2004). Target selectivity in EF-hand calcium binding proteins. Biochim Biophys Acta.

[B40] Nelson MR, Thulin E, Fagan PA, Forsen S, Chazin WJ (2002). The EF-hand domain: a globally cooperative structural unit. Protein Sci.

[B41] Nelson MR, Chazin WJ (1998). An interaction-based analysis of calcium-induced conformational changes in Ca2+ sensor proteins. Protein Sci.

[B42] Hill A (1910). The combinations of haemoglobin with oxygen and with carbon monoxide. J Physiology.

[B43] Adair G (1925). The hemolglobin system. The oxygen dissociation curve of hemoglobin. J Biol Chem.

[B44] Monod J, Wyman J, Changeux JP (1965). On the Nature of Allosteric Transitions: A Plausible Model. J Mol Biol.

[B45] Porumb T (1994). Determination of calcium-binding constants by flow dialysis. Anal Biochem.

[B46] Weiss JN (1997). The Hill equation revisited: uses and misuses. Faseb J.

[B47] Haiech J, Kilhoffer MC (2002). Deconvolution of calcium-binding curves. Facts and fantasies. Methods Mol Biol.

[B48] Guillou JL, Nakata H, Cooper DM (1999). Inhibition by calcium of mammalian adenylyl cyclases. J Biol Chem.

[B49] Shifman JM, Choi MH, Mihalas S, Mayo SL, Kennedy MB (2006). Ca2+/calmodulin-dependent protein kinase II (CaMKII) is activated by calmodulin with two bound calciums. Proc Natl Acad Sci USA.

[B50] Kung C, Preston RR, Maley ME, Ling KY, Kanabrocki JA, Seavey BR, Saimi Y (1992). In vivo Paramecium mutants show that calmodulin orchestrates membrane responses to stimuli. Cell Calcium.

[B51] Aloy P, Russell RB (2006). Structural systems biology: modelling protein interactions. Nat Rev Mol Cell Biol.

[B52] Stemmer PM, Klee CB (1994). Dual calcium ion regulation of calcineurin by calmodulin and calcineurin B. Biochemistry.

