# Supplementary material for: Elucidating the mechanisms of cooperative calcium-calmodulin interactions: a structural systems biology approach
Source: BMC Syst Biol. 2008 Jun 2;2:48. doi: 10.1186/1752-0509-2-48 (PMC2435525; doi:10.1186/1752-0509-2-48)
Supplement: Additional file 1 — The relationship between the Adair and independent binding models. The relationship between macroscopic constants derived from the Adair equation and the proposed Ca2+-CaM binding model with free Ca2+ access. [file 1752-0509-2-48-S1.pdf]

Additional File 1

The relationship between macroscopic constants derived from the Adair equation and the proposed Ca^{2+}-CaM binding model with free Ca^{2+} access

Here we establish the link between the mathematical models for the modified Adair equation and the Ca^{2+}-CaM-specific binding model that allows non-sequential free access and release of ions. We compare the Adair equation with the proposed model and derive the relationship between the macroscopic affinity constants that can be obtained by applying the two models.

In order to characterize the Ca^{2+}-CaM interaction properties, recently published data from calcium binding experiments have been fit with the Hill and Adair equations [1-8]. In particular, the modified Adair equation [9] for flow dialysis-derived Ca^{2+} binding data was used to calculate macroscopic dissociation constants [3]. However, the “dumbbell” shape of CaM molecule with two pairs of EF-hand Ca^{2+} binding sites suggests that the ions appear to have free access to these sites at any given time. The probability of binding depends on the dissociation constant of each site, but can change when CaM undergoes a conformational transition due to ion binding at other sites or interaction with another protein. We sought to compare the difference in consecutive binding (the modified Adair equation) and CaM structure-observed free Ca^{2+} binding and release from EF-hand loops (Equation 2 in Materials and Methods).

The amount of Ca^{2+} bound to CaM according to the sequential binding mechanism (the modified Adair model) is given by Equation (1) in [3] (without the term for non-specific binding):

$$y = \frac{a \cdot x + 2 \cdot b \cdot x^2 + 3 \cdot c \cdot x^3 + 4 \cdot d \cdot x^4}{1 + a \cdot x + 2 \cdot b \cdot x^2 + 3 \cdot c \cdot x^3 + 4 \cdot d \cdot x^4}, \quad (1)$$

where y and x are the bound and free Ca^{2+} concentrations, respectively, and $a = \frac{1}{K_{AK1}}$, $b = \frac{1}{K_{AK1} \cdot K_{AK2}}$, $c = \frac{1}{K_{AK1} \cdot K_{AK2} \cdot K_{AK3}}$, $d = \frac{1}{K_{AK1} \cdot K_{AK2} \cdot K_{AK3} \cdot K_{AK4}}$. K_{AK1}, K_{AK2}, K_{AK3}, K_{AK4} are the macroscopic Ca^{2+} dissociation constants for CaM obtained from the experimental Ca^{2+}-CaM binding curve using the Adair model.

Multiplication of both the numerator and denominator by $K_{AK1} \cdot K_{AK2} \cdot K_{AK3} \cdot K_{AK4}$ transforms (1) into the following equation:

$$y = \frac{K_{AK2} \cdot K_{AK3} \cdot K_{AK4} \cdot x + 2 \cdot K_{AK3} \cdot K_{AK4} \cdot x^2 + 3 \cdot K_{AK4} \cdot x^3 + 4 \cdot x^4}{K_{AK1} \cdot K_{AK2} \cdot K_{AK3} \cdot K_{AK4} + K_{AK2} \cdot K_{AK3} \cdot K_{AK4} \cdot x + 2 \cdot K_{AK3} \cdot K_{AK4} \cdot x^2 + 3 \cdot K_{AK4} \cdot x^3 + 4 \cdot x^4}. \qquad (2)$$

The amount of Ca^{2+} bound to a protein with four Ca^{2+} free access binding sites is given by:

$$y = \frac{x}{x + K_{ind1}} + \frac{x}{x + K_{ind2}} + \frac{x}{x + K_{ind3}} + \frac{x}{x + K_{ind4}}, \qquad (3)$$

where K_{ind1}, K_{ind2}, K_{ind3}, K_{ind4} are the macroscopic Ca^{2+} dissociation constants from CaM obtained from the experimental Ca^{2+}-CaM binding curve using the model for a multisite protein with free Ca^{2+} access to EF-hand binding sites.

The comparison of the denominators of the nonsequential binding and the Adair models reveals the following relationship between macroscopic constants:

$$\begin{aligned}
&K_{ind1} + K_{ind2} + K_{ind3} + K_{ind4} = K_{AK4} \\
&K_{ind1} \cdot K_{ind2} + K_{ind1} \cdot K_{ind3} + K_{ind1} \cdot K_{ind4} + K_{ind2} \cdot K_{ind3} + K_{ind2} \cdot K_{ind4} + K_{ind2} \cdot K_{ind4} = K_{AK3} \cdot K_{AK4} \\
&K_{ind1} \cdot K_{ind2} \cdot K_{ind3} + K_{ind1} \cdot K_{ind2} \cdot K_{ind4} + K_{ind1} \cdot K_{ind3} \cdot K_{ind4} + K_{ind2} \cdot K_{ind3} \cdot K_{ind4} = K_{AK2} \cdot K_{AK3} \cdot K_{AK4} \\
&K_{ind1} \cdot K_{ind2} \cdot K_{ind3} \cdot K_{ind4} = K_{AK1} \cdot K_{AK2} \cdot K_{AK3} \cdot K_{AK4}
\end{aligned} . \qquad (4)$$

The system of equations (4) has a rather complex solution. In order to estimate a simple relationship between the two models, we assume that the binding affinities are close to each other. In this case the equations (4) can be presented as:

$$\begin{aligned}
&4 \cdot K_{ind4} \approx K_{AK4} \\
&6 \cdot K_{ind3} \cdot K_{ind3} \approx K_{AK3} \cdot K_{AK3} \\
&4 \cdot K_{ind2} \cdot K_{ind2} \cdot K_{ind2} \approx K_{AK2} \cdot K_{AK2} \cdot K_{AK2} \\
&K_{ind1} \cdot K_{ind1} \cdot K_{ind1} \cdot K_{ind1} \approx K_{AK1} \cdot K_{AK1} \cdot K_{AK1} \cdot K_{AK1}
\end{aligned} \qquad (5)$$

Or more explicitly the relationship between the macroscopic constants from the non-sequential binding and sequential binding models is given by:

$$\begin{aligned}
&K_{ind4} \approx \frac{K_{AK4}}{4} \\
&K_{ind3} \approx \frac{K_{AK3}}{\sqrt{6}} \ . \\
&K_{ind2} \approx \frac{K_{AK2}}{\sqrt[3]{4}} \\
&K_{ind1} \approx K_{AK1}
\end{aligned} \qquad (6)$$

It follows from the relationship (6) that while one of the four macroscopic constants derived using the modified Adair equation [3, 9] is exactly the same as the constant obtained using the multisite binding model with free ligand access, the other three model constants are significantly different. The equilibrium dissociation constants for the second, third and forth binding sites will differ by factors of approximately $\frac{1}{\sqrt[3]{4}}$, $\frac{1}{\sqrt{6}}$, and $\frac{1}{4}$, respectively. This result highlights the effect of different assumptions made to describe a biochemical reaction on the values of the resulting dissociation constants.

References

1. Minowa O, Yagi K: **Calcium binding to tryptic fragments of calmodulin.** *J Biochem (Tokyo)* 1984, **96:**1175-1182.
2. Peersen OB, Madsen TS, Falke JJ: **Intermolecular tuning of calmodulin by target peptides and proteins: differential effects on Ca2+ binding and implications for kinase activation.** *Protein Sci* 1997, **6:**794-807.
3. Mirzoeva S, Weigand S, Lukas TJ, Shuvalova L, Anderson WF, Watterson DM: **Analysis of the functional coupling between Calmodulin's calcium binding and peptide recognition properties.** *Biochemistry* 1999, **38:**14117-14118.
4. Grabarek Z: **Structure of a trapped intermediate of calmodulin: calcium regulation of EF-hand proteins from a new perspective.** *J Mol Biol* 2005, **346:**1351-1366.
5. Iida S, Potter JD: **Calcium binding to calmodulin. Cooperativity of the calcium-binding sites.** *J Biochem (Tokyo)* 1986, **99:**1765-1772.
6. Ikura M: **Calcium binding and conformational response in EF-hand proteins.** *Trends Biochem Sci* 1996, **21:**14-17.
7. Porumb T: **Determination of calcium-binding constants by flow dialysis.** *Anal Biochem* 1994, **220:**227-237.
8. Maune JF, Klee CB, Beckingham K: **Ca2+ binding and conformational change in two series of point mutations to the individual Ca(2+)-binding sites of calmodulin.** *J Biol Chem* 1992, **267:**5286-5295.
9. Stemmer PM, Klee CB: **Dual calcium ion regulation of calcineurin by calmodulin and calcineurin B.** *Biochemistry* 1994, **33:**6859-6866.
